# Resuscitation-promoting factor (Rpf) terminates dormancy among diverse soil bacteria

**DOI:** 10.1128/msystems.01517-24

**Published:** 2025-04-16

**Authors:** Jay T. Lennon, Brent K. Lehmkuhl, Lingling Chen, Melissa Illingworth, Venus Kuo, Mario E. Muscarella

**Affiliations:** 1 Department of Biology, Indiana University 123993, Bloomington, Indiana, USA; 2 Department of Molecular and Cellular Biochemistry, Indiana University, Bloomington, Indiana, USA; 3 Institute of Arctic Biology and Department of Biology and Wildlife, University of Alaska 124480, Fairbanks, Alaska, USA; Los Alamos National Laboratory, Los Alamos, New Mexico, USA

**Keywords:** seed bank, persistence, signaling, traits, biodiversity

## Abstract

Microorganisms often inhabit environments that are suboptimal for growth and reproduction. To survive when challenged by such conditions, individuals engage in dormancy, where they enter a metabolically inactive state. For this persistence strategy to confer an evolutionary advantage, microorganisms must be able to resuscitate and reproduce when conditions improve. Among bacteria in the phylum Actinomycetota, dormancy can be terminated by resuscitation-promoting factor (Rpf), an exoenzyme that hydrolyzes glycosidic bonds in the peptidoglycan of cell walls. We characterized Rpf from *Micrococcus* KBS0714, a bacterium isolated from agricultural soil. The protein exhibited high substrate affinity *in vitro*, even though resuscitation was maximized in live-cell assays at micromolar concentrations. Site-directed mutations at conserved catalytic sites significantly reduced or eliminated resuscitation, as did the deletion of repeating motifs in a lectin-encoding linker region. We then tested the effects of recombinant Rpf from *Micrococcus* KBS0714 on a diverse set of dormant soil bacteria. Patterns of resuscitation mapped onto strain phylogeny, which reflected core features of the cell envelope. Additionally, the direction and magnitude of the Rpf effect were associated with functional traits, in particular, aspects of the moisture niche and biofilm production, which are critical for understanding dormancy and the persistence of microbial populations in soils. These findings expand our understanding of how Rpf may affect seed bank dynamics with implications for the diversity and functioning of microorganisms in terrestrial ecosystems.

**IMPORTANCE:**

Dormancy is a process whereby individuals enter a reversible state of reduced metabolic activity. In fluctuating environments, dormancy protects individuals from unfavorable conditions, enhancing fitness and buffering populations against extinction. However, waking up from dormancy is a critical yet risky decision. Some bacteria resuscitate stochastically, while others rely on environmental cues or signals from neighboring cells to transition back to active growth. Resuscitation-promoting factor (Rpf) is an exoenzyme that cleaves bonds in the peptidoglycan of bacterial cell walls, facilitating dormancy termination and enabling regrowth. Although this family of proteins has been well characterized in model organisms and clinically relevant strains, our study characterizes Rpf from a soil bacterium and examines its effects on resuscitation across a diverse collection of bacteria, linking it to functional traits that may influence dormancy dynamics in both natural and managed ecosystems.

## INTRODUCTION

Soils are among the most diverse microbial habitats on Earth (1, 2). A single gram of soil can harbor thousands of co-occurring taxa (3), and there is a substantial turnover in composition across spatial scales (4). One life-history strategy that contributes to soil biodiversity is dormancy, a process that allows individuals to enter a reversible state of reduced metabolic activity (5). Only ~1% of the soil microbial biomass pool is metabolically active (6). The remaining viable biomass is a ’seed bank.’ This reservoir of dormant individuals plays a crucial role in the evolution and persistence of populations, the maintenance of biodiversity, and the overall functioning of ecosystems (7, 8, 9).

Dormancy has independently evolved multiple times, giving rise to a variety of mechanisms by which microorganisms enter and exit metabolic states (10). Dormancy is often initiated in response to changing environmental conditions. For instance, some filamentous cyanobacteria form resting structures, called akinetes, in response to light or phosphorus limitation (11). Fungal spore production is initiated in part by hormonal signaling between arbuscular mycorrhizae and the plant host (12). Additionally, some bacteria enter dormancy by forming metabolically inactive persister cells, which can be generated stochastically or in response to adverse environmental conditions (13).

Resuscitation is also crucial for the success of dormancy as a survival strategy. Since awakening at an inopportune time can be maladaptive, microorganisms often rely on cues to time their resuscitation. This decision-making process is typically regulated by the detection of nutrients through membrane-bound receptors (14) or, in some cases, by more sophisticated mechanisms, such as tracking electrochemical potentials, a form of microbial memory that can be used to gauge environmental quality (15). Another example comes from the Actinomycetota, a phylum of bacteria that emerge from dormancy using an extracellular enzyme known as resuscitation-promoting factor (Rpf). The ’scout hypothesis’ proposes that these bacteria may stochastically awaken and secrete Rpf into the environment, thereby triggering the resuscitation of neighboring cells (16).

Rpfs were first identified in populations of *Micrococcus luteus*. When late stationary-phase cultures were exposed to filter-sterilized supernatants from actively growing cells, dormant bacteria rapidly resumed their metabolic activity (17). Subsequently, the growth-stimulating compound was identified as a small (∼16 kDa) protein with muralytic activity (18). Rpf cleaves β-(1,4) glycosidic bonds in peptidoglycan, a major constituent of the cell wall in nearly all bacteria (19, 20). In addition to facilitating the remodeling and turnover of dormant cell walls necessary for outgrowth, the muropeptides released during Rpf-mediated hydrolysis may act as specialized signaling molecules that awaken closely related bacteria (21).

Rpf is broadly distributed among diverse lineages of the Actinomycetota (22, 23). It is clinically relevant because Rpf can terminate latency in *Mycobacterium* strains, including those responsible for tuberculosis (24). Rpf is also found among environmental isolates (25) and may be particularly important in soils. Metagenomic analyses indicated that up to 20% of soil genomes may contain Rpf homologs (5). Meanwhile, experimental evidence suggests that Rpf has direct and indirect effects on seed bank dynamics. The addition of Rpf to soil influenced bacterial and fungal structure, which was associated with altered respiration and plant performance (26). Beyond these observations, little is known about how Rpf from soils affects diverse strains of bacteria.

In this study, we conducted experiments with recombinant Rpf generated from an actinomycetal strain, *Micrococcus* KBS0714, that was isolated from agricultural soil (27, 28). Using purified protein, we measured enzyme kinetics to assess the catalytic potential and substrate affinity of the novel Rpf. After evaluating its concentration dependence and potential for inhibition, we examined the Rpf’s effect on the resuscitation dynamics in a previously characterized collection of soil bacteria (27, 29), aiming to determine the spectrum of activity across phylogenetic groups and its relationship to functional traits involved in soil dormancy.

## RESULTS

### Rpf is a high-affinity enzyme

**Fig 1:**
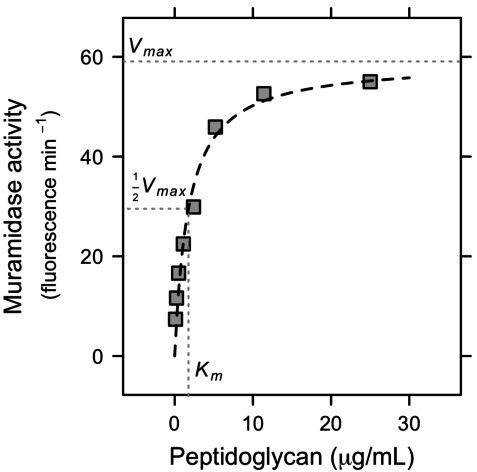
Enzyme kinetics of Rpf. We constructed a Michaelis–Menten curve (see equation 1) to characterize the catalytic properties of purified Rpf from the soil bacterium *Micrococcus* KBS0714. We estimated $V_{\max}$, the maximum catalytic rate under substrate-saturating conditions, and $K_{m}$, the half-saturation constant that reflects the enzyme’s substrate affinity.

We quantified the enzyme kinetics of purified recombinant Rpf from *Micrococcus* KBS0714 (Fig. S1) using fluorescein-labeled peptidoglycan (Fig. 1). When hydrolyzed, the fluorescent substrate products can be used as a measure of muramidase activity. We characterized Rpf activity using the Michaelis–Menten function (see equation 1). Using maximum likelihood methods, we estimated that $V_{\text{max}}$ was 59 units of fluorescein ${\text{min}}^{-1}$, and that $K_{m}$ was 1.8 mg of peptidoglycan mL ${\text{min}}^{-1}$. The relatively low $K_{m}$ is consistent with reports of Rpf having high affinity and the capacity to catalyze reactions at low substrate concentrations (18).

### The Rpf effect on growth is concentration-dependent

**Fig 2:**
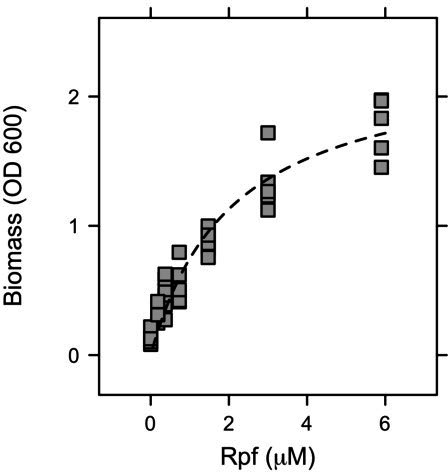
Concentration dependence of Rpf on bacterial growth. To assess the stimulatory and inhibitory effects of Rpf on bacterial resuscitation, dormant *Micrococcus* KBS0714 cells were exposed to varying concentrations of the recombinant protein, and the resulting biomass data were fit to the Monod growth equation.

To characterize any stimulatory or inhibitory effects of Rpf, we measured the biomass of *Micrococcus* KBS0714 following the incubation of dormant cells in fresh medium with different concentrations of recombinant protein (0–6 µM). We fit the resulting data with the Monod growth model, which mathematically is identical to that of the Michaelis–Menten equation (see equation 1). Growth monotonically increased with Rpf concentration (Fig. 2). The half-saturation constant (*K_s_*) was 2.1 µM Rpf, and the estimated maximum biomass (OD_600_) was 2.3 (Fig. 2). Based on this relationship, there was no evidence for growth inhibition of *Micrococcus* KBS0714 at the elevated Rpf concentrations tested in our study. See Fig. S2 for discussion on negative controls.

### Rpf mutations disrupted resuscitation

**Fig 3:**
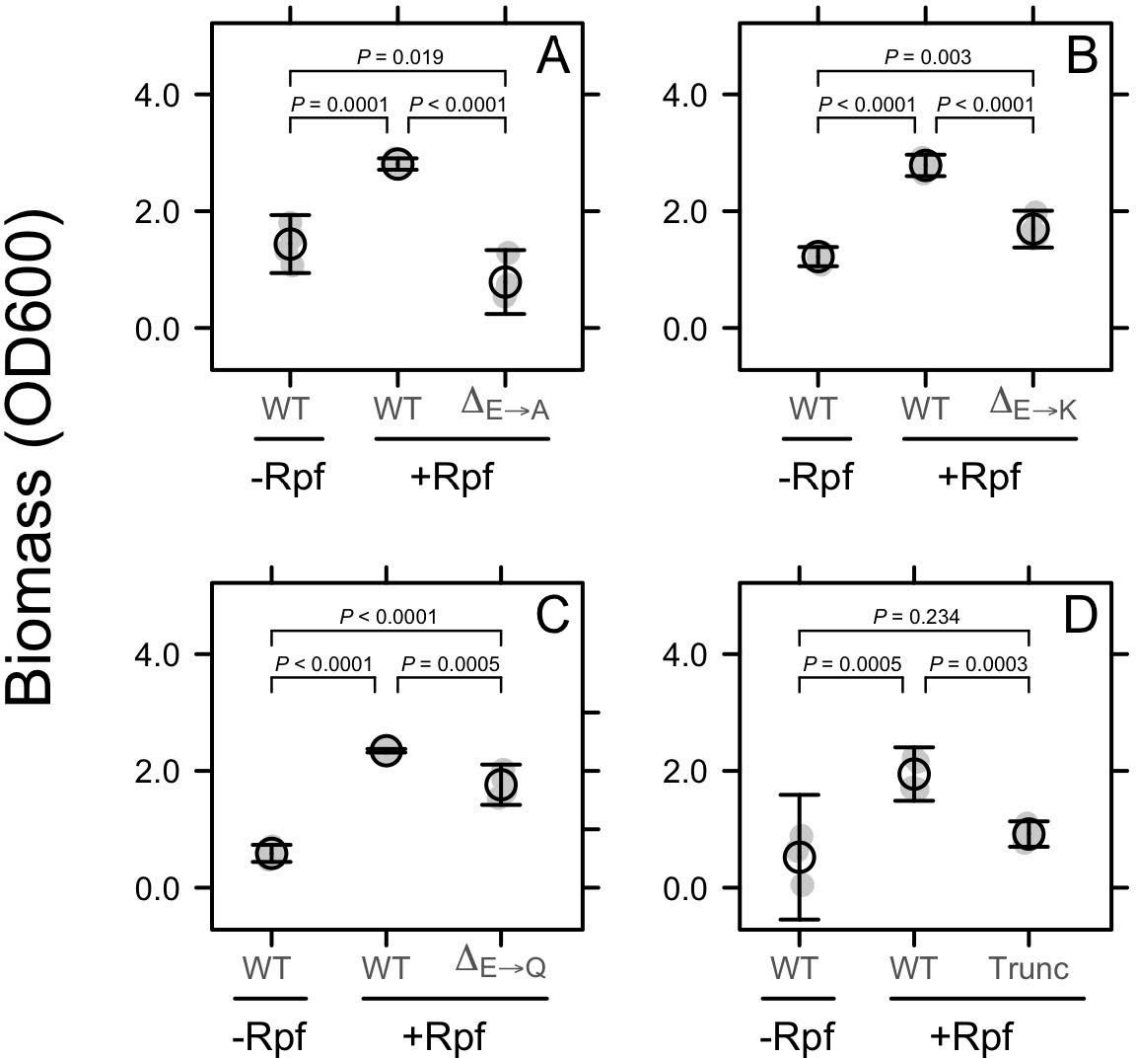
Rpf mutations disrupted resuscitation. Effects of site-directed and truncation mutations on growth of *Micrococcus* KBS0714. Gray symbols represent raw data. Black symbols represent the mean ± standard error of the mean (*n* = 4). In panels A–C, we used site-directed mutagenesis to change a conserved catalytic site (E54) from a glutamic acid residue (E) to an alanine (A), lysine (K), or glutamine (Q) residue. In panel D, we show results from a truncation mutation that deleted repeating motifs in a lectin-encoding linker region along with the adjacent LysM domain. *P*-values are associated with one-way ANOVA, followed by a Tukey’s post-hoc comparison.

When dormant cells were exposed to recombinant protein from the native *rpf* sequence, we observed a two to fourfold increase in biomass (Fig. 3; $P_{\text{adj}}\leq 0.0005$). This effect was diminished by site-directed mutagenesis of the conserved catalytic site (E54) within the *rpf* gene (see Fig. S3). When glutamic acid (E), a charged amino acid, was mutated to alanine (A), a hydrophobic amino acid, there was no effect of recombinant Rpf on biomass (Fig. 3A; $P_{\text{adj}}<0.0001$). However, resuscitation was only reduced by 40% when glutamic acid was mutated to lysine (K), a charged amino acid (Fig. 3B; $P_{\text{adj}}<0.0001$). Similarly, when the glutamic acid residue was mutated to a polar uncharged amino acid, glutamine (Q), resuscitation was reduced by 25% ($P_{\text{adj}}=0.0005$), with biomass still threefold greater than the −Rpf control ($P_{\text{adj}}<0.0001$; Fig. 3C).

We identified structural variation in the amino acid sequence of *rpf* in *Micrococcus* KBS0714 (Fig. S3). Compared to *Micrococcus luteus* (Fleming), which has been used as a model for studying Rpf (18), there are a number of repeating motifs in a lectin-encoding linker region between the lysozyme-like and LysM domains. In *Micrococcus* KBS0714, this linker region contains 21 tandemly arrayed repeats, whereas the Fleming strain contains only three repeats (Fig. S3). To assess whether this structural variation affected resuscitation, we deleted the variable linker region along with the LysM domain of Rpf. The activity of the truncated Rpf was reduced by more than 50% ($P_{\text{adj}}=0.0003$) and eliminated resuscitation based on the fact that biomass was indistinguishable from the −Rpf control ($P_{\text{adj}}=0.234$; Fig. 3D).

### Rpf altered population dynamics

**Fig 4:**
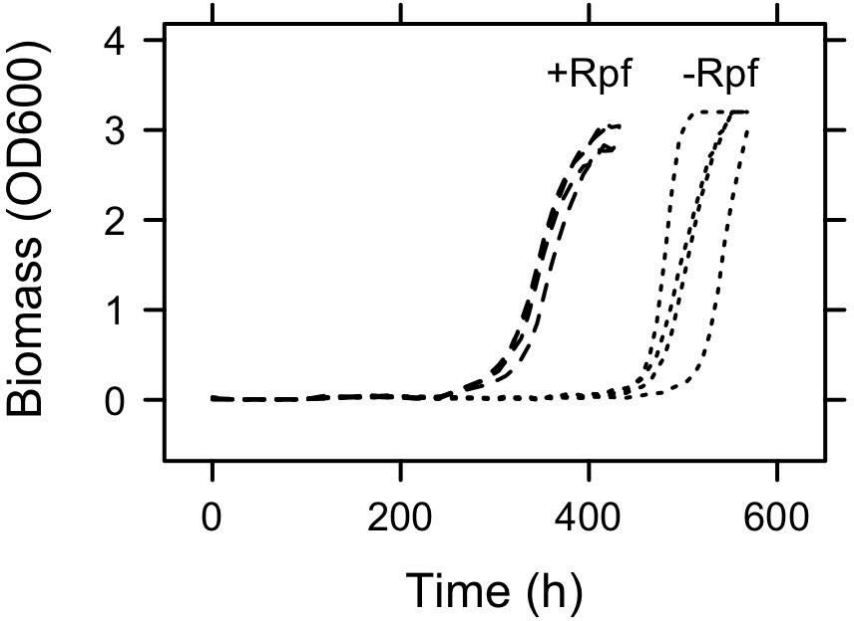
Population dynamics of dormant bacteria exposed to Rpf. Following 90 days of starvation, we transferred dormant *Micrococcus* KBS0714 into fresh medium with or without recombinant Rpf. The addition of Rpf reduced lag time by ∼7.5 days (37%). It also reduced among-population variability in $\mu_{\max}$ and lag time, indicating that Rpf may synchronize resuscitation-related processes.

We quantified the effect of recombinant Rpf on the dynamics of a dormant population of *Micrococcus* KBS0714. After maintaining cells in late stationary phase for 90 days, we transferred cells to fresh medium. The addition of Rpf to these cultures reduced lag time by 37% from 476±27.1 to 298±3.4 h ($t_{3.1}=6.52$, *P* = 0.007, Fig. 4). As a consequence, experimentally resuscitated populations entered exponential growth phase ∼7.5 days earlier than control populations. In contrast, Rpf had no effect on the maximum growth rate ($\mu_{\max}$) (−Rpf = $0.058\pm 0.0213\;{day}^{-1}$; +Rpf = $0.028\pm 0.0003\;{day}^{-1}$, *P* =0.25) or the biomass yield (−Rpf = 4.1±0.27; +Rpf = 3.6±0.17, $t_{5.0}=1.68$, *P* = 0.15) of *Micrococcus* KBS0714. However, Rpf minimized variability among replicate populations in $\mu_{\max}$ $F_{\left(3,3\right)}=0.0002$[, P<0.0001] and lag time [$F_{\left(3,3\right)}=0.016$, P=0.007] amounting to a 5-fold and 30-fold reduction, respectively, in the coefficient of variation (CV) of these growth curve parameters (see Fig. S4).

### Diverse bacteria affected by Rpf

**Fig 5:**
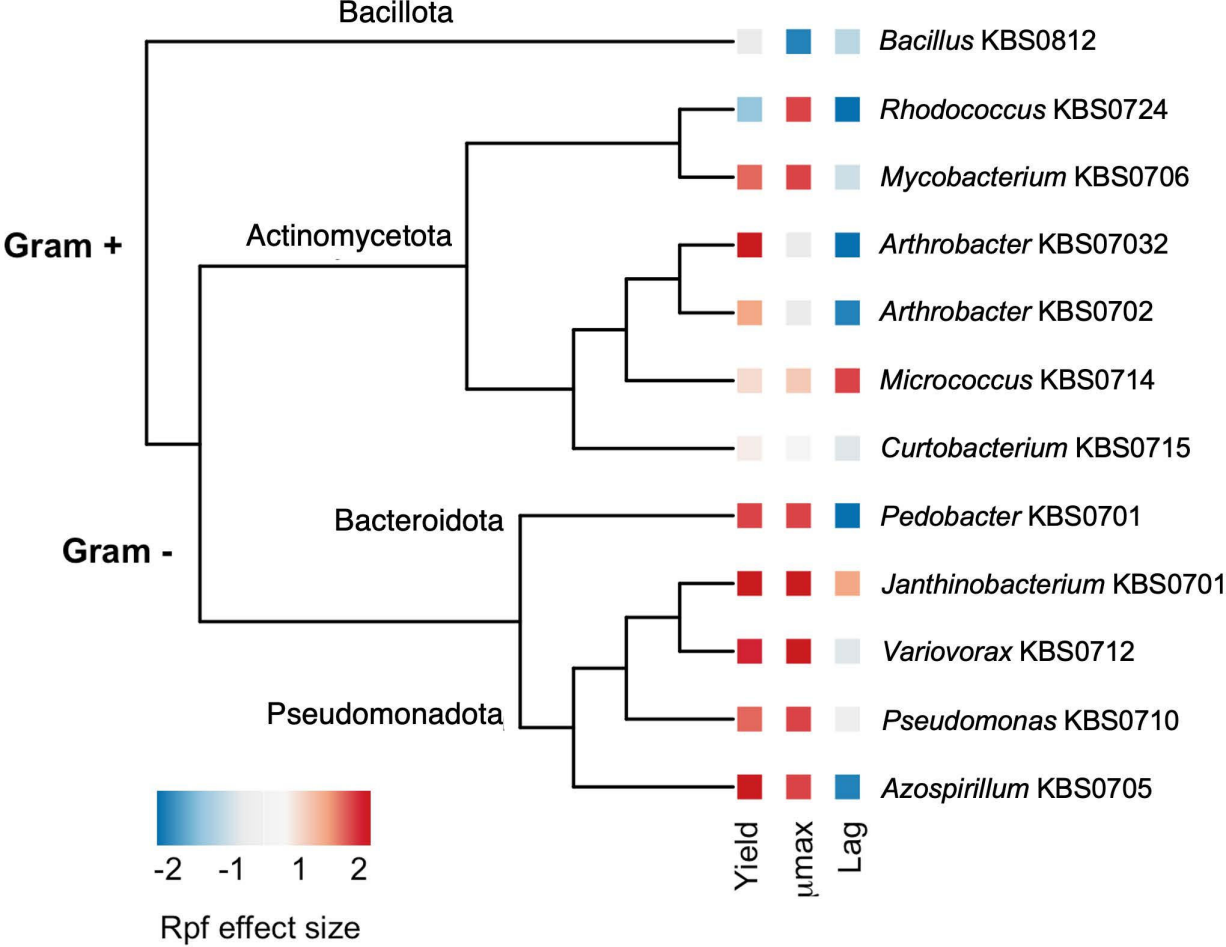
Effects of Rpf on the resuscitation of diverse soil bacteria from dormancy. We mapped the Rpf effect sizes onto a maximum likelihood tree constructed from the 16S rRNA gene sequences for 12 strains of soil bacteria from a culture collection (27). For each strain of dormant bacteria, we characterized resuscitation based on growth curve parameters (i.e., $\mu_{\text{max}}$, yield, and lag time) in the presence (+Rpf) and absence (−Rpf) of recombinant protein. With that information, we calculated the Rpf effect size for each growth curve parameter and mapped it onto the tree.

We used a well-characterized collection of soil bacteria (27) to assess how dormant cells with different evolutionary histories respond to Rpf. We transferred dormant cells into fresh medium with and without recombinant Rpf from *Micrococcus* KBS0714. During the resuscitation process, we quantified growth curve parameters (see equation 2) and calculated the standardized Rpf effect size for each of the 12 strains of soil bacteria. There was a significant interaction between strain and Rpf treatment on growth yield [$F_{\left(1,11\right)}=3.35$, *P* = 0009] and $\mu_{\text{max}}$ [$F_{\left(1,11\right)}=4.07$, *P* = 0.0001] as determined using two-way analysis of variance (ANOVA) (Fig. S5). Some of this variation could be explained by the evolutionary relationship of strains used in our study (Table S2). For example, we detected phylogenetic signal for the Rpf effect size on $\mu_{\text{max}}$ (Pagel’s λ: 1.0, *P* = 0.013; Blomberg’s *K*: 0.99, *P* = 0.018), suggesting that aspects of resuscitation were consistent with a model of Brownian motion based on a maximum likelihood tree constructed from 16S rRNA gene sequences (Fig. 5).

In addition, variation in resuscitation among bacterial strains was associated with key features of the cell envelope. Specifically, gram-positive strains responded differently to Rpf than gram-negative strains (Fig. 5). Using a generalized linear mixed model, we detected a significant interaction between these cell types (random effect) and the Rpf treatment (fixed effect) on growth yield [$F_{\left(1,82\right)}=9.47$, *P* = 0.003] and $\mu_{\text{max}}$ [$F_{\left(1,82\right)}=7.30$, *P* = 0.008]. Rpf had a 3.2- and 6.2-fold greater effect size on $\mu_{\text{max}}$ and growth yield, respectively, for Gram-negative bacteria compared to Gram-positive bacteria. In contrast, the Rpf effect size for lag time was similar for bacteria with contrasting cell types (Fig. 5).

**Fig 6:**
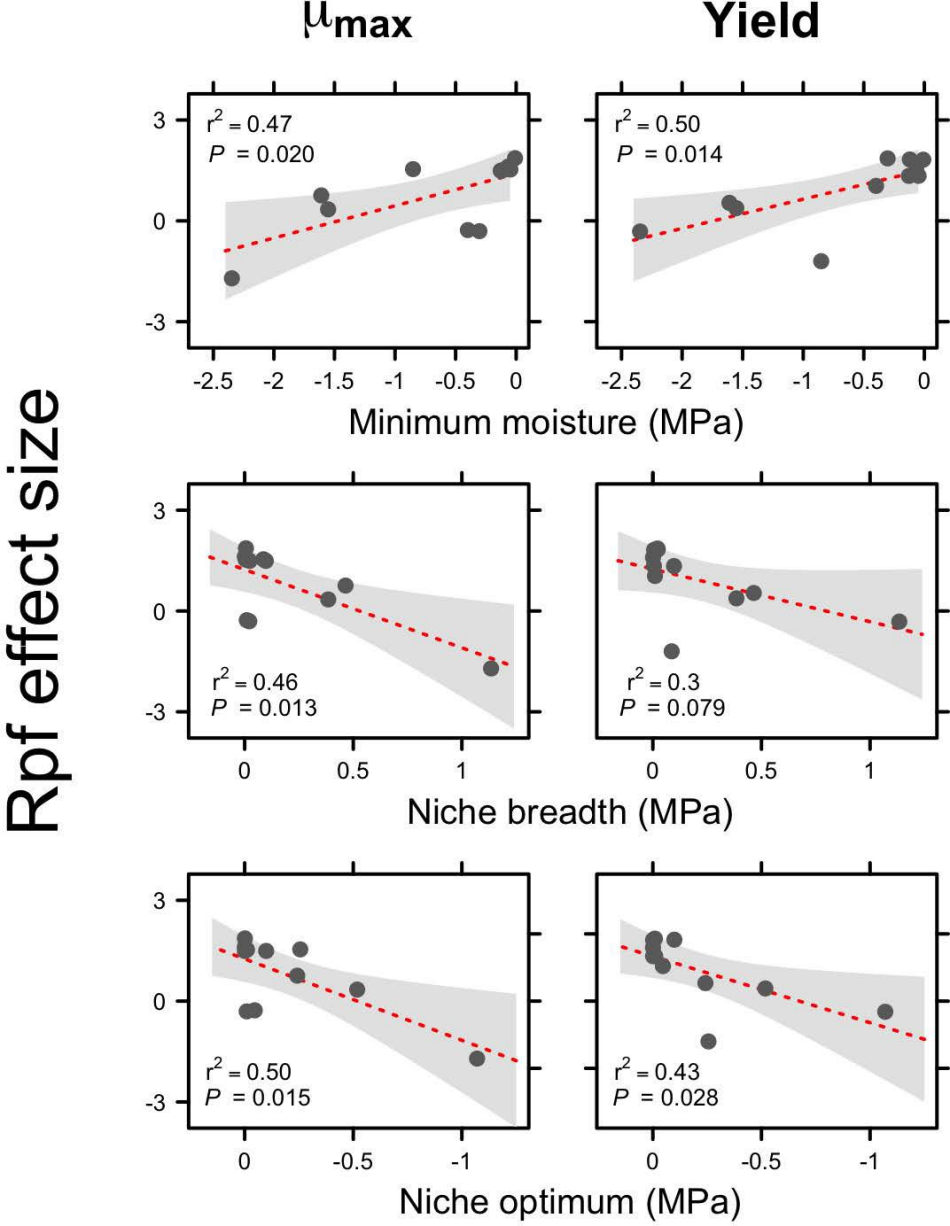
Relationship between Rpf effect size and functional traits. Resuscitation of dormant bacteria via Rpf was associated with a strain’s moisture niche. For each strain from a well-characterized collection of soil bacteria (27), we transferred dormant cells to fresh medium in the presence (+Rpf) and absence (−Rpf) of recombinant protein. We then quantified resuscitation dynamics using parameters from the growth curve function (see equation 2). With that information, we calculated the Rpf effect size for each growth curve parameter (i.e., $\mu_{\text{max}}$, yield, and lag time) and related it to different features of the moisture niche, namely, the minimum water potential ($W_{\text{min}}$), the niche breadth (b), and the niche optimum ($W_{\text{opt}}$) (27).

Lastly, by leveraging functional trait data from the culture collection (27), we determined whether variation in resuscitation could be explained by physiological characteristics that were measured on individual strains (Table S3). In particular, resuscitation was related to features of the moisture niche, which was characterized based on the respiratory activity of each strain along a gradient of soil water potential (MPa) (27). We found that the Rpf effect size for both $\mu_{\text{max}}$ and growth yield increased linearly with the minimum water potential ($W_{\text{min}}$), a quantity that defines the soil moisture content, where respiration (R) is equal to 5% of maximum respiration ($R_{\max}$). We also found that the Rpf effect size for both $\mu_{\text{max}}$ and lag time decreased linearly with the niche breadth (b) and optimum water potential ($W_{\text{opt}}$) of the bacterial moisture niche (Fig. 6). The Rpf effect size for lag time was not directly related to features of the moisture niche, but was positively correlated with biofilm production [$F_{\left(1,9\right)}=6.08$, *P* = 0.036, $r^{2}=0.40$], which can confer tolerance to desiccation stress (30).

## DISCUSSION

Dormancy is a widespread survival strategy among microorganisms, enabling them to persist in harsh and fluctuating environments (5). It has independently evolved across the tree of life, reflecting variation in the mechanisms that govern entry into and exit from dormancy (10). Among bacteria in the phylum Actinomycetota, dormancy can be terminated by resuscitation-promoting factors, exoenzymes that hydrolyze glycosidic bonds in the peptidoglycan of the bacterial cell wall. Although much of what is known about Rpf stems from studies on *Micrococcus luteus* and *Mycobacterium tuberculosis*, Rpf is present in many other Actinomycetota species and potentially in other phyla (22). In this study, we characterized Rpf from *Micrococcus* KBS0714, a bacterial isolate from agricultural soil (27, 28). We evaluated the effects of recombinant Rpf from this actinomycetal strain on a diverse set of dormant soil bacteria (Fig. 5) previously characterized by their functional genomics and traits (27, 29). Patterns of resuscitation were related to the phylogeny of these bacteria and could be explained in part by conserved features of the cell envelope. Additionally, responses to Rpf were associated with the microbial moisture niche, which is critical for understanding persistence and resuscitation during soil dormancy. These findings enhance our understanding of how dormancy affects the diversity and functioning of terrestrial ecosystems.

### Kinetics and catalysis of Rpf

Rpf was initially characterized as a bacterial cytokine due to its small molecular size and its potent ability to resuscitate dormant cultures of *Micrococcus luteus* at picomolar concentrations (10^−12^ M) (18). In contrast, we observed resuscitation at micromolar (10^−6^ M) concentrations, consistent with responses reported for other environmental isolates (25). While some of these concentration-dependent effects may reflect the maximum rate of peptidoglycan hydrolysis ($V_{\text{max}}$), it is also important to consider substrate affinity when characterizing enzyme kinetics. To this end, we determined that Rpf from *Micrococcus* KBS0714 has a low half-saturation constant ($K_{m}$), indicating strong binding to peptidoglycan, even at low substrate concentrations (Fig. 1), which could be advantageous in heterogeneous soil environments. A comparative analysis of Rpf kinetics awaits further studies, as similar experiments have yet to be conducted with other bacteria.

While most studies emphasize its capacity to stimulate growth, Rpf also has the potential to be inhibitory. Similar to lysozyme, Rpf cleaves the β-(1,4) linkage between N-acetylglucosamine and N-acetylmuramic acid in peptidoglycan. This breaking of glycosidic bonds initiates hydrolysis, facilitating cell wall remodeling necessary for growth and division. However, Rpf could lead to excessive bond disruption or interference with regulatory processes involved in biosynthesis that compromise cell wall integrity. Consistent with this, viable cell densities decreased by 25% when *Rhodococcus* was exposed to 1,000 picomolar Rpf compared to 1 picomolar Rpf (31). We observed no evidence of such inhibition in our live cell assays (Fig. 2). Instead, the growth of *Micrococcus* KBS0714 monotonically increased with Rpf concentration up to 6 μM (Fig. 2). Inhibition may emerge at higher Rpf levels, but whether such concentrations are ecologically relevant in soil environments remains unclear.

### Genetic conservation of Rpf function

Resuscitation was strongly affected by site-directed mutations in conserved regions of the Rpf gene. The catalytic domain of Rpf found toward the N-terminal contains residues that are essential for muralytic activity (Fig. S3). Mutations in the glutamic acid residue (E54) significantly reduced or eliminated the ability of recombinant Rpf to resuscitate dormant *Micrococcus* KBS0714 (Fig. 3). These findings are consistent with genetic studies across diverse bacterial strains, demonstrating that the conserved glutamate residue (E54) in Rpf is essential for peptidoglycan hydrolysis (24, 20, 32).

Resuscitation was also affected by mutations in other regions of the protein sequence, specifically the repeating motifs in a lectin-encoding linker region and the LysM domain. These repeats likely arose from tandem duplication due to replication slippage, which could have consequences for Rpf function. Lectins are involved in carbohydrate binding and can modify cell–substrate interactions. Deletion of these lectin repeats, along with the LysM domain, eliminated the resuscitation of dormant *Micrococcus* KBS0714, highlighting the functional significance of these repeating motifs. The additional lectins in Rpf may enhance peptidoglycan binding capacity and contribute to its high substrate affinity (Fig. 1). Thus, our findings lend support to the view that there are conserved catalytic sites that are essential for resuscitation (e.g., E54). They also suggest that structural variation (e.g., lectin repeats), in addition to the LysM domain, may be important for carbohydrate binding, which together may influence resuscitation in physically complex environments like soil.

### Population dynamics of resuscitated bacteria

One of the hallmarks of Rpf is that it reduces the lag time of dormant populations after waking up from dormancy (33). The duration of lag time reflects the activity of cells and is influenced by the time required to carry out biosynthetic processes, such as the production of RNA and proteins that are necessary for growth and reproduction. Consistent with other reports, our experiments revealed that Rpf can significantly reduce lag time. For example, Rpf from a *Tomitella* (Mycobacteriales) isolate reduced lag time by nearly 60% from 12 to 5 days (25). Following 90 days of starvation, we documented that a one-time addition of recombinant Rpf allowed dormant *Micrococcus* KBS0714 to resuscitate 1 week earlier than control populations (Fig. 4). In an ecological context, accelerated growth responses could have major consequences for populations living in diverse communities, like soil, where there are rapid fluctuations in environmental conditions that affect microbial metabolism (34). If Rpf enables dormant *Micrococcus* KBS0714 to resuscitate days ahead of other dormant bacteria, it could promote persistence, even in the presence of less responsive but potentially faster-growing competitors. This type of lottery effect may be further enhanced by reducing the stochastic nature of resuscitation (16, 35). For instance, we observed that the among-population variation in both $\mu_{\max}$ and lag time across replicate populations was significantly reduced (Fig. 4), indicating that Rpf may synchronize resuscitation-related processes and minimize random events that might otherwise disrupt regrowth of the population.

### Rpf in a community context

At any given time, most microorganisms in soil are slow-growing or metabolically inactive (5, 6). The reactivation of dormant cells may be facilitated by Rpf. While Rpf is predominantly produced by bacteria within the phylum Actinomycetota, its target substrate, peptidoglycan, is a structural component of the cell wall that is found in nearly all bacteria. Additionally, the glycosidic bond cleaved by Rpf is chemically invariant, suggesting that a wide variety of dormant bacteria within the soil seed bank may be susceptible to resuscitation by this exoenzyme. Our results indicate that diverse taxa respond to Rpf but not uniformly. Parameters, such as yield and $\mu_{\max}$, showed greater sensitivity to Rpf exposure, whereas lag time was less affected (Fig. 5; Fig. S5).

Variation in resuscitation could be attributed in part to the evolutionary history of the strains used in our study. For example, there was a strong phylogenetic signal associated with the response of $\mu_{\max}$ to Rpf. One likely factor contributing to this phylogenetic signal is the structural differences in the cell envelope, particularly the distribution and amount of peptidoglycan found among strains (Fig. 5; Fig. S5). In Gram-positive bacteria, approximately 90% of the cell wall consists of peptidoglycan, forming a thick layer that is directly exposed to the external environment (36). In contrast, Gram-negative bacteria have a thinner peptidoglycan layer, accounting for only 10% of the cell wall, which is protected between the inner and outer membranes (36). Because of these differences, we expected that Rpf would have a stronger effect on Gram-positive bacteria. In contrast, our results indicate that Rpf from *Micrococcus* KBS0714 had a stronger positive effect on yield and $\mu_{\max}$ in Gram-negative bacteria. Such findings suggest that other mechanisms besides direct contact between enzyme and substrate (peptidoglycan) may be important for the resuscitation of dormant bacteria by Rpf. For example, Rpf acts synergistically with Rpf-interacting protein A (RipA), an endopeptidase that cleaves stem peptides involved in peptidoglycan cross-linking. RipA localizes at the septa of growing cells, facilitating cell wall digestion and releasing small muropeptides, which may serve as signaling molecules, thereby imparting specificity to the resuscitation process (37, 38). Although more work is needed to characterize the spectrum and mechanisms of enzymatic activity among diverse microbial taxa, the cell envelope is a coarse-grained trait that may be useful for making predictions about Rpf-mediated resuscitation in complex communities.

The response of diverse bacterial strains to Rpf may be influenced by other functional traits. In soils, microbial activity is often governed by moisture availability. When water content is low—due to precipitation, soil texture, or evapotranspiration—microbial activity typically drops, causing cells to enter a dormant state. When water content suddenly increases, for example, following a rain event, microorganisms often exhibit pulses of metabolic activity as they exit dormancy (34). Understanding how microorganisms respond to environmental fluctuations and how this regulates transitions into and out of dormancy has important implications for the diversity and function of soil microbiomes.

We found that the response of dormant cells to Rpf was related to a bacterial strain’s moisture niche (27). Specifically, dry-adapted strains with a narrow niche breadth were less responsive to Rpf, while wet-adapted strains with a broader moisture niche breadth were more responsive to Rpf (Fig. 6). Another potential adaptation to low moisture conditions is biofilm production (30). Our results revealed a positive correlation between the Rpf effect on lag time and biofilm production, suggesting that a smaller investment into biofilms may allow strains to resuscitate more readily. This could indicate a trade-off or reflect differences in enzyme activity and diffusion within the exopolymeric matrix.

The relationships between resuscitation and functional traits (Fig. 6) warrant further investigation and validation. One approach would be to examine the distribution and diversity of *rpf* genes in soil metagenomes. While early studies attempted to quantify *rpf* genes in contrasting ecosystems (5), the availability of larger databases and more advanced computational tools (39) now enables independent testing of the trait relationships identified in our study without the constraints of cultivation-based approaches. These data could also support the development of niche models incorporating environmental variables (40) to assess whether dormancy strategies are more prevalent in habitats experiencing moisture fluctuations or prolonged drought. Such insights would advance a predictive, macroecological framework for microbial dormancy (41).

Although Rpf is a widespread mechanism that regulates bacterial dormancy, other factors contribute to microbial seed bank dynamics (8). Rpf-mediated resuscitation operates alongside other dormancy mechanisms, such as endosporulation and persister cell formation, which may determine how a bacterium interprets and responds to its environment. As a result, microbes can exist in shallow or deep states of dormancy (42) while possessing other traits that influence longevity and responsiveness to internal and external cues. Collectively, these processes influence species interactions and shape the microbial seed bank, creating structure and memory that can lead to emergent phenomena (8). Our findings contribute to understanding dormancy regulation and its role in microbial diversity across habitats, such as soils, with implications for indirect effects and feedbacks that influence ecosystem stability and function (26).

## MATERIALS AND METHODS

### Strains and culturing

We used *Micrococcus* KBS0714, an actinomycetal strain that was isolated from agricultural soil (27). It shares 99% sequence similarity in the 16S rRNA gene with *M. luteus* NCTC 2662, a model organism for studying Rpf (18). The genomic characteristics and physiology of KBS0714 have been described in detail elsewhere (27, 28). For routine culturing, we maintained KBS0714 in R2A broth at 25°C on an orbital shaker (150 rpm). Unless otherwise stated, we induced dormancy by allowing cells to enter stationary phase, and then keeping them on a shaker table (150 rpm) for 30 days, followed by static (non-shaken) conditions for an additional 60 days. Prior to using these dormant bacteria for resuscitation experiments, we washed the cells by pelleting and resuspending them five times in phosphate-buffered saline (PBS; pH = 7.0) to remove residual medium.

### Recombinant protein expression

We amplified and cloned the *rpf* gene of *Micrococcus* KBS0714 into an *Escherichia coli* expression host to produce recombinant Rpf. As described in greater detail elsewhere (26), we extracted genomic DNA from KBS0714 using a Microbial DNA Isolation Kit (MoBio). We then amplified the open reading frame of the *rpf* gene using two primers, Upper‐F 5′ GCC CAT ATG GCC ACC GTG GAC ACC TG 3′ and Lower‐R 5′ GGG GAT CCG GTC AGG CGT CTC AGG 3′, which incorporated the EcoRI restriction sites NdeI (forward primer) and BamHI (reverse primer) (18, 43). The PCR conditions are as follows: initial: 95°C for 5 min, 30 cycles of 95°C for 30 s, 55°C for 30 s, 72°C for 1 min, and final extension at 72°C for 7 min. The PCR product was confirmed using gel electrophoresis and Sanger sequencing (26). We performed an NdeI/BamHI restriction digest on the 850 bp *rpf* gene amplicon and ligated the restriction product into a pET15b expression vector with an N-terminus polyhistidine-tag (pET15B-His6-rpf). We then transformed the recombinant expression plasmid into the *E. coli* Origami BL21 (DE3) expression host (*E. coli* pET15b-His6-rpf).

To overexpress Rpf, we grew *E. coli* pET15b-His6-rpf in 1 L of lysogeny broth (LB) and induced protein expression at an OD_600_ of ~0.6 with isopropyl β-D-1-thiogalactopyranoside (IPTG) (final concentration 0.1 mM). We then lysed the cells via sonication, which was followed by centrifugation and filtration (0.45 µm). With this preparation, we purified the recombinant Rpf protein with the N-terminus polyhistidine tag via Ni-NTA Purification (Invitrogen) using a 10 mL gravity-fed column with a 2 mL resin bed. The protein was run through the column twice and rinsed with 3× volume of wash Ni-buffer A (300 mM NaCl, 50 mM Tris-HCl, 5 mM imidazole) followed by elution with 125 mM imidazole elution buffer in 1 mL fractions. Rpf was further purified via buffer exchange using 10 mL Zeba Spin Desalting Columns (Thermo Fisher) with protein buffer (20 mM Tris-HCl, 100 mM NaCl) according to manufacturer’s instructions followed by 0.2 µm syringe filtration. Overexpression and protein purity were confirmed by SDS-PAGE and Western blotting (Fig. S1). Protein concentrations were determined with Bradford assays (Invitrogen).

### Enzyme kinetics

We quantified the enzymatic activity of recombinant Rpf with the EnzChek Lysozyme Assay Kit (Molecular Probes). The assay uses cell walls from *Micrococcus lysodeikticus* that are extensively labeled with fluorescein. Enzymatic cleavage of the β-(1,4) glycosidic bond relieves quenching, which yields a fluorescence signal that is proportional to lysozyme activity. We incubated Rpf (1 mg/mL, 34 µM) over a range of substrate concentrations (0–30 µg/L) at 22°C for 24 h. As a control, we deactivated the enzyme via incubation at 90°C for 1 h. Fluorescence was then measured with a BioTek Synergy H1 plate reader using excitation/emission wavelengths of 485/530 nm. We quantified the enzyme kinetics by fitting the data with a Michaelis–Menten function using maximum likelihood procedures (44):


(1) $$V=\frac{V_{\text{max}}\cdot S}{K_{m}+S}$$

where V is the rate of peptidoglycan hydrolysis, $V_{\text{max}}$ is the maximum rate of peptidoglycan hydrolysis, $K_{m}$ is the half-saturation constant, and S is the substrate concentration.

### Concentration dependence of Rpf

We characterized the growth of *Micrococcus* KBS0714 in response to a concentration gradient of recombinant Rpf. After growing *Micrococcus* KBS0714 to late stationary phase (30 days), dormant cells were washed 5X in PBS and then transferred into wells of a Corning Costar flat-bottom 48-well culture plates (Thermo Fisher) with 1 mL R2A broth. Recombinant protein was added to replicate wells (*n* = 4) to achieve final concentration of Rpf ranging from 0 to 6 µM. Following 120 h of incubation, we estimated biomass as optical density (OD_600_), acknowledging that absorbance values may vary due to differences in cell size, abundance, and physiology. We fit the resulting data to a Monod growth equation, which is identical in form to the Michaelis-Menten function used for characterizing enzyme kinetics for Rpf (see equation 1).

### Population dynamics

We used a growth curve assay to quantify the resuscitation dynamics of *Micrococcus* KBS0714 in response to a one-time addition of recombinant Rpf. After being maintained in long-term stationary phase for 90 d, we washed dormant bacteria 5× in PBS before inoculating them into 250 mL side-arm flasks containing 35 mL of lactate minimal medium with 1% L-lithium lactate (45). In quadruplicate (*n* = 4), the flasks were amended with recombinant Rpf (0.5 µM final concentration) or a protein buffer as a control (see Fig. S2). We measured biomass (OD_600_) over time using a Biophotometer (Eppendorf) while the flasks were incubated on a shaker (150 rpm) at 25°C. We fit the resulting data to a modified Gompertz equation (46) using maximum likelihood procedures:


(2) $$Y=b_{0}+A\cdot \exp\left\{-\exp\left[\frac{\mu_{\text{max}}\cdot e}{A}(L-t)+1\right]\right\}$$

where $\mu_{\text{max}}$ is the maximum growth rate (h^${}^{-1}$^), λ is lag time (h), A is the carrying capacity or biomass yield (OD_600_), and $b_{0}$ is the intercept. We tested for the effect of the Rpf treatment on the growth parameters using *t*-tests. We also quantified the within-treatment CV of the growth curve parameters and used *F*-tests to assess whether Rpf treatment altered the equality of variances.

### Rpf mutations

We created mutations in the *rpf* gene of *Micrococcus* KBS0714 to better understand the mechanisms of Rpf activity. First, we mutated the conserved glutamate residue (E54) of Rpf using the QuikChange site-directed mutagenesis kit (Stratagene) following the manufacturer’s instructions. See Table S1 for conditions and reagents used for mutagenesis, including primer sets, master mix recipe, and PCR conditions. We methylated the parental DNA by incubating with 1 µL of DpnI enzyme at 37°C for 1 h. With the resulting mutated DNA, we transformed competent *E. coli* (Invitrogen) using 5 µL of PCR product to generate a population of transformants that grew on LB plates containing 100 µg/mL of ampicillin. We grew these cells for ∼16 h in 5 mL of LB with the appropriate antibiotics on a shaker (150 rpm) at 37°C. We then extracted plasmids from the cells using the QIAprep Spin Miniprep Kit following the manufacturer’s instructions. We confirmed the success of the mutagenesis via Sanger sequencing (47). Second, we truncated a variable linker and LysM domain of the native *rpf* gene using PCR amplification, cloning, transformation, and Rpf expression, as described in the previous sections. Specifically, two primers, rpf1-F 5′ACC GCG ACC GTG CAG CGC TAG GAT CCG 3′ and trncrpf-R 5′CGG ATC CTA GCG CTG CAC GGT CGC GGT 3′ were designed to PCR amplify the N-terminus lysozyme-like region omitting the variable linker and LysM domain. The PCR resulted in a ∼375 bp amplicon as determined by gel electrophoresis and Sanger sequencing. We then overexpressed and purified the recombinant protein for each of the mutant sequences following the procedures described above. Dormant cells were washed 5× in PBS prior to being incubated in 13 mm test tubes with 2 mL of R2A broth containing 2.5 µM Rpf on a shaker table (150 rpm) at 25°C for 72–360 h. Growth was estimated as endpoint biomass (OD_600_) on replicate (*n* = 4) test tubes using a Biophotometer (Eppendorf). We compared growth of *Micrococcus* KBS0714 exposed to Rpf (native vs. mutated) and the negative control using one-way ANOVA and Tukey’s HSD.

### Diverse bacterial responses to Rpf

We evaluated the specificity of Rpf by exposing different strains of dormant bacteria to the recombinant protein that was expressed from the *rpf* gene in *Micrococcus* KBS0714. We used 12 chemoorganoheterotrophs that are part of a genomically and physiologically well-characterized culture collection (see Tables S2 and S3) (27, 29). Gram-negative bacteria included *Pedobacter* KBS0701, *Azospirillum* KBS0705, *Pseudomonas* KB0710, *Janthinobacterium* KBS0711, and *Variovorax* KBS0712. Gram-positive bacteria included *Arthrobacter* KBS0702, *Arthrobacter* KBS0703, *Mycobacterium* KBS0706, *Micrococcus* KBS0714, *Curtobacterium* KBS0715, *Rhodococcus* KBS0724, and *Bacillus* (KBS0812). Each strain was grown in batch culture containing R2A broth on a shaker (150 rpm) at 25°C. After being maintained in long-term stationary phase for 30 days, we washed the dormant cells 5× in PBS and dispensed them into replicate wells (*n* = 4) of Corning Costar flat-bottom 48-well culture plates (Thermo Fisher) with 1 mL R2A broth that contained 0.5 µmol/L recombinant protein (+Rpf) or an equal volume of 100 mM Tris-HCl protein buffer (−Rpf). We then measured growth as optical density (OD_600_) using a BioTek plate reader (Thermo Fisher) at 25°C under constant shaking (150 rpm). With the resulting data, we estimated growth curve parameters by fitting a modified Gompertz model (see equation 2). With these parameters, we calculated the Rpf effect size for each strain using Cohen’s D = $\frac{{\bar{x}}_{\text{rpf}}-{\bar{x}}_{\text{control}}}{\text{sd}}$, where $\bar{x}$ is the mean value of a growth parameter, and sd is the pooled standard deviation across treatment levels.

We evaluated the effect of Rpf with respect to the evolutionary history of the bacterial strains. With existing 16S rRNA gene sequences (27, 29), we created a maximum likelihood tree with RAxML that used a general time reversible model with the Gamma model of rate heterogeneity. We mapped the Rpf effect size for growth curve parameters ($\mu_{\text{max}}$, lag time, and biomass yield) onto the phylogenetic tree using the ape package in R (48). We then tested for phylogenetic signal of the Rpf effect size using Blomberg’s K and Pagel’s λ with the phytools package in R (49).

We evaluated the effect of Rpf in relation to functional traits that were previously measured on the bacterial strains (27). These traits included biofilm production, motility, and microaerotolerance (Table S3). In addition, we characterized the moisture niche of each strain by measuring rates of respiration along a gradient of soil water potential (MPa) (Table S3). Using maximum likelihood methods, we extracted niche parameters from fitted data, including minimum moisture ($W_{\text{min}}$), optimal moisture ($W_{\text{opt}}$), and moisture breadth (b). We then used simple linear regression to describe relationships between functional traits and the Rpf effect size.

## Data Availability

Code and data to reproduce all analyses are available on GitHub (https://github.com/LennonLab/Rpf) and Zenodo (10.5281/zenodo.15104488).

## SUPPLEMENTAL MATERIAL

Supplemental Material msystems.01517-24-s0001.pdf Supplemental tables and figures.

ASM does not own the copyrights to Supplemental Material that may be linked to, or accessed through, an article. The authors have granted ASM a non-exclusive, world-wide license to publish the Supplemental Material files. Please contact the corresponding author directly for reuse.
