# Supplementary material for: Resuscitation-promoting factor (Rpf) terminates dormancy among diverse soil bacteria
Source: mSystems. 2025 Apr 16;10(5):e01517-24. doi: 10.1128/msystems.01517-24 (PMC12090717; doi:10.1128/msystems.01517-24)
Supplement: Supplemental Material — Supplemental tables and figures. [file msystems.01517-24-s0001.pdf]

Supplementary Information: Resuscitation-promoting factor (Rpf) terminates dormancy among diverse soil bacteria

Jay T. Lennon[1*], Brent K. Lehmkuhl[1], Lingling Chen[2], Melissa Illingworth[2], Venus Kuo[1], Mario Muscarella[3]

1 Department of Biology, Indiana University, Bloomington, IN, 47405, USA

2 Department of Molecular and Cellular Biochemistry, Indiana University, Bloomington, IN, 47405, USA

3 Institute of Arctic Biology & Department of Biology and Wildlife, University of Alaska, Fairbanks, Alaska, USA

* corresponding author: lennonj@iu.edu

TABLE S1 **Experimental conditions for Rpf mutagenesis.** Upper: primer sets and sequences used for site-directed mutagenesis of the conserved glutamate residue in *rpf* from *Micrococcus* KBS0714. Capitalized bases indicate introduced mutations in the sequence. Lower: reagents for master mix used in PCR for site-directed mutagenesis.

**Primers**	**Sequence**
5′ Rpf E54A	cctcgccgagtgcGCGtccagcggcacc
3′ Rpf E54A	ggtgccgctggaCGCgcactcggcgagg
5′ Rpf E54Q	cctcgccgagtgcCAGtccagcggcacc
3′ Rpf E54Q	ggtgccgctggaCTGgcactcggcgagg
5′ Rpf E54K	cctcgccgagtgcAAGtccagcggcacc
3′ Rpf E54K	ggtgccgctggaCTTgcactcggcgagg

**Reagent**	**Volume (μL)**
H_2O	35
10X Phusion HF buffer	5
100% DMSO	4
25 mM dNTPs	1
50 mM $MgCl_2$	1
5′ Rpf mutagenesis primer, 4 μM	1
3′ Rpf mutagenesis primer, 4 μM	1
Rpf template plasmid, 15 ng/μL	1
Phusion enzyme	1
**Total**	**50**

TABLE S2 Bacterial taxonomy. We measured the growth characteristics of different soil bacteria after exposure to recombinant Rpf. More information about the enrichment, isolation, and genomics of these strains can be found elsewhere [1, 2].

**Strain**	**Cell type**	**Phylum**	**Family**	**Genus**
KBS0701	Gram -	Bacteroidota	Sphingobacteriaceae	*Pedobacter*
KBS0702	Gram +	Actinomycetota	Micrococcaceae	*Arthrobacter*
KBS0703	Gram +	Actinomycetota	Micrococcaceae	*Arthrobacter*
KBS0705	Gram -	Pseudomonadota	Azospirillaceae	*Azospirillum*
KBS0706	Gram +	Actinomycetota	Mycobacteriaceae	*Mycobacterium*
KBS0710	Gram -	Pseudomonadota	Pseudomonadaceae	*Pseudomonas*
KBS0711	Gram -	Pseudomonadota	Oxalobacteraceae	*Janthinobacterium*
KBS0712	Gram -	Pseudomonadota	Comamonadaceae	*Variovorax*
KBS0714	Gram +	Actinomycetota	Micrococcaceae	*Micrococcus*
KBS0715	Gram +	Actinomycetota	Microbacteriaceae	*Curtobacterium*
KBS0724	Gram +	Actinomycetota	Nocardiaceae	*Rhodococcus*
KBS0812	Gram +	Bacillota	Bacillaceae	*Bacillus*

TABLE S3 **Bacterial traits.** Functional traits of soil bacteria that were exposed to recombinant Rpf. In a previous study [1], the moisture niche was constructed for each strain by exposing cells to a soil moisture gradient and measuring rates of respiration (R). From this, we estimated the maximum respiration rate (R_{max}, μg C-CO_2 g^{-1} d^{-1}), the optimum water potential (W_{opt}, MPa), niche breadth (b, MPa), and minimum water potential (W_{min}, MPa). Separately, we measured microaerotolerance (O_2-Tol, proportion of biomass produced under low O_2 [2%] to biomass produced under high O_2 [21.5%]), maximum growth rate (μ_{max}, h^{-1}), lag time (h), biofilm production (a_{550}), and motility (mm/hr).

**Strain**	R_{max}	W_{opt}	b	W_{min}	**O_2-Tol**	μ_{max}	**lag**	**biofilm**	**motility**
KBS0701	59	-0.0007	0.0007	-0.0659	0.52	0.0777	5.41	0.17	0.21
KBS0702	64	-0.0464	0.0097	-0.3986	0.20	0.1151	4.26	0.11	0.42
KBS0703	70	-0.0093	0.0211	-0.3034	0.37	0.1184	4.38	0.19	0.21
KBS0705	74	-0.0990	0.0225	-0.1198	0.34	0.0318	7.01	0.21	0.25
KBS0706	46	-0.0106	0.0048	-0.0477	0.11	0.0325	18.1	1.99	0.25
KBS0710	59	-0.0010	0.0973	-0.1277	1.02	0.0830	6.26	0.53	2.75
KBS0711	64	-0.0010	0.0058	-0.0093	0.23	0.1062	6.47	1.76	1.04
KBS0712	11	-0.2420	0.4645	-1.6092	0.31	0.0526	29.2	2.64	0.17
KBS0714	108	-0.5180	0.3848	-1.5525	0.31	0.0544	7.09	1.01	0.29
KBS0715	92	-0.2560	0.0866	-0.8515	0.73	0.0742	11.14	1.20	0.33
KBS0724	36	-1.0700	1.1345	-2.3469	0.44	0.0182	1.95	3.06	2.00
KBS0812	59	-0.0007	0.0007	-0.0659	0.52	0.0777	5.41	0.17	0.21

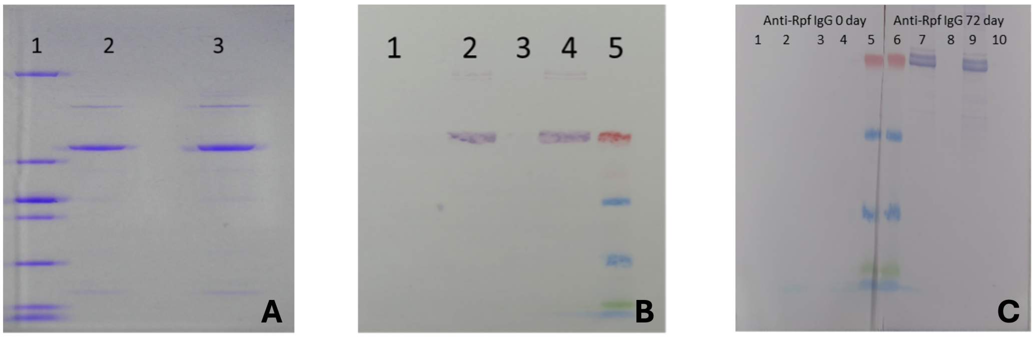

FIG S1 **Rpf over-expression gels. A.** SDS-PAGE of pET15b Rpf at 900 µg/mL confirming protein purity extracted from NI-NA column extraction. First lane is a low-range protein marker (100, 30, 25, 20, 15, 10, 5, 3.4 kDa), second lane is 5 µL of Rpf, third lane is 10 µL of Rpf. **B.** Western blot of 5 and 10 µL of pET15b recombinant Rpf at 900 µg/mL concentration bound with anti-Histidine (1:50000) primary IgG in lane 2 and 4. Lane 1 and 3 are empty. Lane 5 is a low-range western marker (red/40, blue/15, green/10, blue/2.6, blue/1.7 kDa). **C.** Western blot of 5 and 10 µL of pET15b Rpf protein at 900 µg/mL concentrations bound with 72 day Rpf-specific primary IgG antibodies (1:50000) in lanes 7 and 9. This is contrasted with the 0 day antibodies which did not bind to Rpf protein in lane 2 and 4. Lanes 1, 3, 8, and 10 are empty. Lanes 5 and 6 are low-range western marker (red/40, blue/15, green/10, blue/2.6, blue/1.7 kDa)

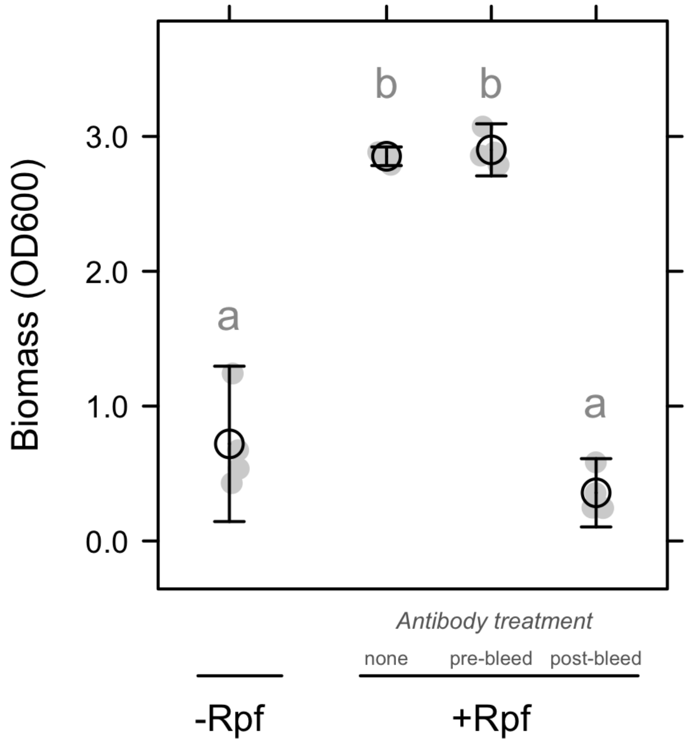

FIG S2 **Assessment of negative controls.** We performed an experiment where we measured the regrowth of dormant *Micrococcus* KBS0714 in R2A broth medium with and without Rpf. Final biomass (OD_{600}) was estimated at 120 h. In the negative control (-Rpf), we only added protein buffer. For the +Rpf treatment, cells were exposed to 1.6 µM of recombinant Rpf. In the +Rpf treatment, cells were assigned to an antibody treatment. They were either left untreated ("none") or exposed to antibodies from rabbit serum (600 µg/mL) collected before (day 0, "pre-bleed") or after (day 28, "post-bleed") the Rpf antigen was administered. Results demonstrate that antibody binding to Rpf active site of muralytic activity is sufficient for eliminating resuscitation effects on *Micrococcus* KBS0714 biomass to levels comparable to protein buffer

control. Treatments sharing the same letter are not significantly different (one-way ANVOA followed by Tukey's HSD, $P > 0.05$).

```
                           *
Fleming      1   ATVDTWDRLA  ECESNGTWDI  NTGNGFYGGV  QFTLSSWQAV  GGEGYPHQAS
                 ||||||||||  ||||||||||  ||||||||||  ||||||||||  ||||||||||
KBS0714      1   ATVDTWDRLA  ECESSGTWDI  NTGNGFYGGV  QFTLSSWQAV  GGEGYPHQAS


Fleming     51   KAEQIKRAEI  LQDLQGWGAW  PLCSQKLGLT  QADADAGDVD  ATEAAPVAVE
                 ||||||||||  ||||||||||  ||||||||||  ||||||||||     |||||||
KBS0714     51   KAEQIKRAEI  LQDLQGWGAW  PLCSQKLGLT  QADADAGDVD     AAPVAVE


Fleming    101   RTATVQR---  ----------  ----QSAADE  AAAEQA----  ----------
                 ||||||                      ||||:|  ||:|||
KBS0714    101   RTATVQRGSQ  SAADETAADQ  AAAEQAAADQ  AAAEQAAADQ  AAAERWAAKQ
                                                                    †

Fleming    151   ----------  ----------  ----------  ----------  ----------

KBS0714    151   AAADQAAAER  WAAKQAAAEQ  AAADKAAAQR  AAAAEKAAAQ  KAAAAEKAAA


Fleming    201   ----------  ---------A  AAAEQAVVAE  AETIVVKSGD  SLWTLANEYE
                                      |  ||||||||||  ||||||||||  |||·||||||
KBS0714    201   QKAAAAEKAA  AQKAAAAEQA  AAAEQAVVAE  AETIVVKSGD  SLWKLANEYE


Fleming    251   VEGGWTALYEA  NKGAVSDAAV  IYVGQELVLP  QA
                 |||||||||||  ||||||||||  ||||||||||  ||
KBS0713    251   VEGGWTALYEA  NKGAVSDAAV  IYVGQELVLP  QA
```

FIG S3 **Amino acid sequences of Rpf.** Amino acid sequences for resuscitation promoting factors (Rpf) from *Micrococcus luteus* (Fleming) and a soil isolate used in this study, *Micrococcus* KBS7014. Starting at residue 42, there is a conserved glutamic acid (E) residue at position 54 (*), which is within the lysozyme-like domain highlighted in green. We made recombinant Rpf with KBS014 strain that had site-directed substitutions at the conserved site to evaluate effects on resuscitation (see Fig. 3 in main text). The lectin-rich linker region, the length of which is greatly reduced in the Fleming strain (-), is highlighted in blue while the LysM domain is highlighted in yellow. We made a recombinant Rpf from *Micrococcus* KBS0714 where a significant fraction of the lectin-rich linker region and the LysM domain starting at residue 145 (†) were truncated. Other symbols follow UniProt convention where solid vertical lines represent fully conserved residues, colons (:) represent residues with strongly

similar properties, and periods (.) represent residues with weakly similar properties.

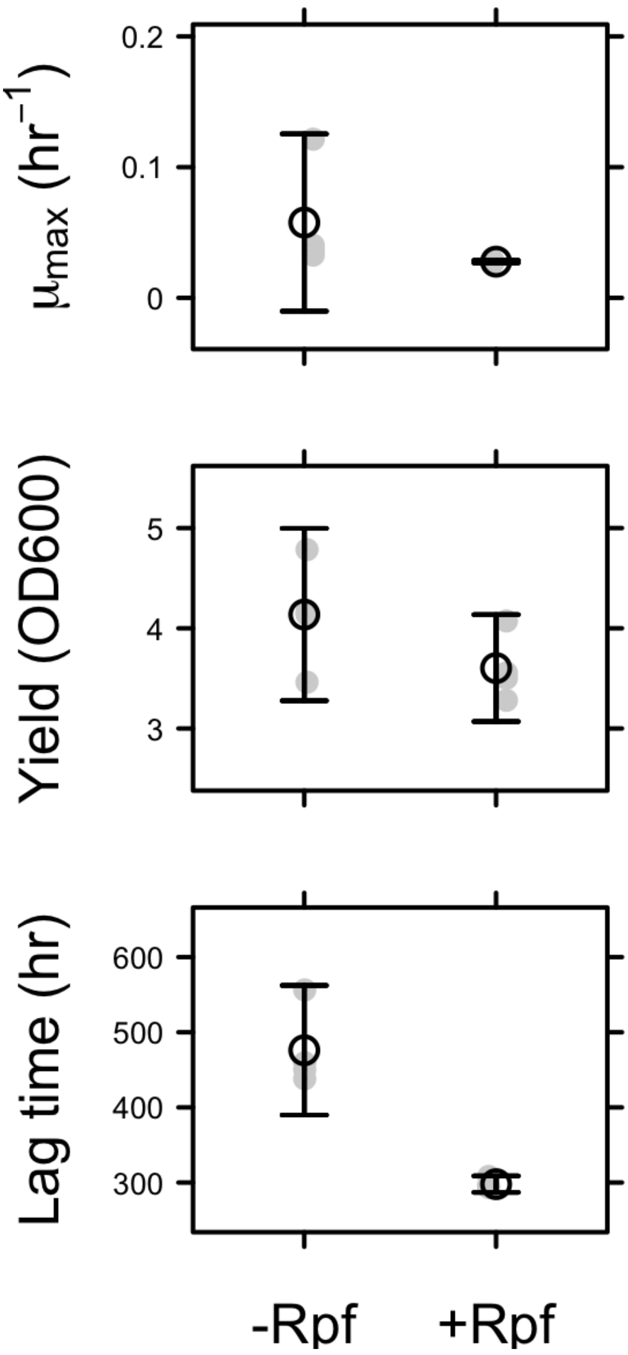

FIG S4 **Growth parameters of *Micrococcus* KBS0714 exposed to Rpf.** We estimated μ_{max}, yield, and lag time after transferring 90-d starved

cells into fresh medium with vs. without recombinant Rpf. Grey symbols represent raw data. Black symbols represent the mean ± SEM (n = 4). See Fig. 4 in main text.

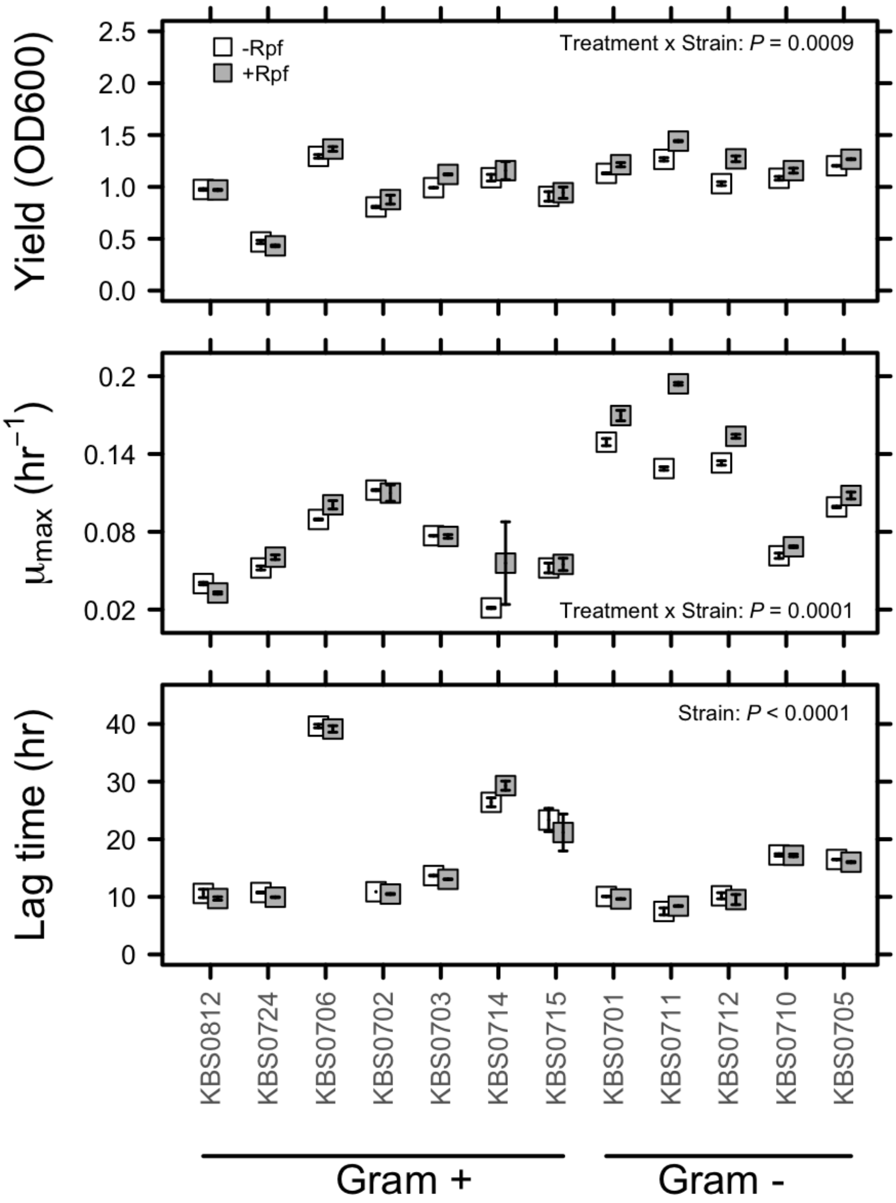

FIG S5 **Growth parameters of diverse bacteria exposed to Rpf.** Growth parameters for different soil bacterial when exposed to recombinant protein (+Rpf) or a negative control (-Rpf). Grey and white symbols represent raw data. Black symbols represent the mean ± SEM (n = 4). Tests of significance were determined using a generalized linear mixed

model (GLMM) where Rpf treatment was a fixed effect and cell type (Gram + vs. Gram -) was a random effect.

REFERENCES

References

[1] **Lennon JT, Aanderud ZT, Lehmkuhl BK, Schoolmaster Jr DR**. 2012. Mapping the niche space of soil microorganisms using taxonomy and traits. *Ecology* 93 (8):1867–1879. doi:https://doi.org/10.1890/11-1745.1.

[2] **Shoemaker WR, Jones SE, Muscarella ME, Behringer MG, Lehmkuhl BK, Lennon JT**. 2021. Microbial population dynamics and evolutionary outcomes under extreme energy-limitation. *Proceedings of the National Academy of Sciences of the United States of America* 118 (e2101691118). doi:10.1073/pnas.2101691118.
